# Hormonal Carcinogenesis in Canine Mammary Cancer: Molecular Mechanisms of Estradiol Involved in Malignant Progression

**DOI:** 10.3390/ani11030608

**Published:** 2021-02-26

**Authors:** Cristian G. Torres, María P. Iturriaga, Pamela Cruz

**Affiliations:** 1 Laboratory of Biomedicine and Regenerative Medicine, Department of Clinical Sciences, Faculty of Veterinary and Animal Sciences, Universidad de Chile, Santiago 8820808, Chile; pamela.cruz.espinoza@gmail.com; 2 Faculty of Veterinary Medicine and Agronomy, Universidad de Las Americas, Santiago 7500975, Chile; pacitaia@gmail.com

**Keywords:** hormonal carcinogenesis, canine mammary cancer, estradiol, tumor progression

## Abstract

**Simple Summary:**

Canine mammary cancer corresponds to a tumor disease that frequently affects female dogs, especially those that are reproductively intact. This disease can decrease the survival and quality of life of affected animals; therefore, it is important to know the underlying mechanisms. Hormonal factors are very important for the development of this pathology. Estradiol, a hormone produced primarily in the ovaries actively participates in the process of mammary tumor formation, but the principal mechanisms behind this carcinogenic role remain incompletely understood. This review will discuss how estradiol would induce its pro-tumor effect on the mammary tissue, thus contributing to the understanding of the mechanisms involved in the development of this disease.

**Abstract:**

Mammary cancer is a frequent neoplasia in female dogs, in which most important risk factors are hormonal. Sexual hormones as estradiol play an important role in mammary carcinogenesis, being able to induce carcinogenic initiation, promotion and progression. However, the molecular mechanisms involved are incompletely understood. Estradiol is synthesized mainly in the ovaries, nevertheless, high concentrations of estradiol and some of its hormonal precursors have also been described in malignant mammary tumor tissue. The mechanisms of action of estradiol include the classic genomic effects that modulate gene transcription, and non-genomic effects, which trigger quick effects after estradiol binds to its specific receptors. These responses modulate various intracellular signaling pathways, triggering post-translational modification of several proteins. This review will discuss the well-known underlying mechanisms associated with the action of estradiol in the malignant progression of canine mammary tumors.

## 1. Canine Mammary Cancer

Canine mammary tumors (CMT) are neoplasms of frequent occurrence in veterinary medicine [1], accounting for approximately 25–70% of all cancers affecting intact female dogs [2,3]. The main risk factors described for this pathology are advanced age (mean 9.2–10.4 years) [4], breed [5,6], intact reproductive status, ovariohysterectomy after 2.5 years old, treatment with progestogens and estrogens, and obesity at early age [7]. In fact, the reproductive endocrine status influences the development of mammary tumors, especially ovarian hormone exposure in the first two years of life [1], which is verified in female dogs undergoing ovariohysterectomy at an early age, especially before the first estrus, where a significant reduction in the risk of mammary cancer occurs. Surgical sterilization at late ages seem not to modify this risk [8,9], however, it does improve survival in mastectomized bitches with estradiol-dependent mammary tumors [10]. This pathology is frequently detected in certain breeds, suggesting a genetic basis. Nevertheless, many of these alterations remain unknown and are currently under study [6,11]. It has been described mutations in the Breast Cancer 1 and 2 (BRCA 1/2) tumor suppressor genes in female dogs with mammary tumors, suggesting their participation in the mammary carcinogenic process [5,11,12]. Recently, an extensive whole-exome and transcriptomic study determined the presence of several somatic mutations in CMTs, where mutations in phosphatidylinositol 3-kinase (*PI3KCA*) oncogene and aberrations in the PI3K-Akt signaling pathway stand out [13]. Regarding estrogens and genetic profile of this disease, there is scarce information related to estradiol receptor α (ERα) gene (*ESR1*) [14]. Preliminary, variations of *ESR1* gene have been described in low aggressive mammary tumors. Thus, animals that exhibit allele for single nucleotide polymorphisms (SNPs) rs397512133, rs397510462, rs851327560 and rs397510612 developed tumors later than wild type dogs, which would suggest that variants of this gene could modify the risk of CMTs [14].

Approximately 50–70% of CMTs are classified histologically as malignant [15,16], implying the potential to invade or metastasize to adjacent tissue [7,9]. However, a recent study has reported only 15% of malignancy [17], outcome likely associated with early diagnosis.

These tumors are very heterogeneous, and about 90% correspond to carcinomas [18,19,20]. The options for treatment are limited to surgery, chemotherapy and, in occasional cases, radiotherapy; however, only surgery has curative potential and is considered the gold-standard treatment for most cases [21].

Clinical and pathological factors with prognostic value have been established. The former includes the size of the primary tumor (tumors smaller than 3 cm have a better prognosis), and the clinical stage (Stages IV–V have a poor prognosis) [21,22]. Pathological factors include the histological type and grade, lymphovascular invasion and the immunoexpression of ERα. Those mammary tumors associated with a better survival prognosis are complex, simple tubular and simple tubulopapillary carcinomas of low histological grade. Instead, carcinosarcomas, solid carcinomas, and anaplastic carcinomas have a poor prognosis. Carcinomas with presence of tumor cells within peritumoral lymphatic vessels are associated to low survival times [18,19]. On the other hand, a high ERα expression is related with a small size of the primary tumor and a low histological grade, among other clinical-pathological features [23,24,25]. A molecular classification for malignant mammary tumors has been described according to the immunohistochemical co-expression of ERα, epidermal growth factor receptor 2 (EGFR-2/HER-2), and baseline biomarkers such as CK14 and p63. Thus, five categories have been established: luminal A (ERα+/HER-2-), luminal B (ERα+/HER-2+), HER-2 overexpression (ERα-/HER-2+), basal-type (ERα-/HER-2-/basal markers+), and normal-type or triple negative [26,27]. The importance of this subclassification lies in the fact that tumors subclassified as luminal A, turn out to display low histological grades and low rates of lymphatic invasion, while basal-type or triple negative tumors usually shown high histological grades and high lymphatic invasion [26].

Based on the information described, it is reasonable to assume that ERα activated by estradiol (E2) fulfills a relevant function in mammary tumorigenesis and tumor progression.

## 2. E2 and Mammary Cancer

Since the physiological requirements of a hormone are constantly changing in the internal environment, its concentration must be subject to accurate regulation. Thus, when there is a lack of control in hormonal secretion, cellular homeostasis is lost, generating potential alterations that can lead to carcinogenesis [28]. In this regard, some hormones can act as initiators or promoters of neoplastic transformation, stimulating cell proliferation and predisposing to genetic alterations [29].

The development of canine mammary tumors is estradiol-dependent, where many of these patients express high tissue ERα levels [30] or high concentrations of serum E2 (>35 pg/mL) [23,30]. The duration of exposure of mammary gland to estrogens seems key to this biological process. An inverse relation between ERα immunoexpression and histological differentiation and survival time has been widely described. Thus, benign and malignant low-grade tumors are ERα-positive, while those malignant, high-grade tend to be ERα-negative [23,24,25]. The *ESR1* gene expression has a similar pattern than the histological expression. Thus, in high-grade carcinomas, this gene is not expressed either [31]. Complementary, Sorenmo et al. [30] recently described that high serum E2 levels and ERα-positive tumors associate with an overall longer time to metastasis, however, this association occurs only in spayed dogs. In fact, in this work, dogs with low histological grade tumors exhibit higher serum E2 concentrations, which reinforces the idea that E2 promotes a pro-carcinogenic effect in ERα-positive mammary carcinomas. In human breast cancer, the most aggressive tumors do not show the presence of the ERα, which has been attributed to methylation of the CpG island in the promoter of the *ESR1* gene [32]. Accordingly, methylation of the CpG island in the canine *ESR1* gene was studied, comparing normal mammary tissue and malignant tumors. No differences were observed in the proportion of methylation between the groups analyzed [32], therefore, the absence of ERα expression in canine malignant tumors would be induced by causes still unknown. 

On the other hand, E2 seems to induce a protective effect in reproductively intact dogs with ERα-negative tumors, where high E2 levels associate with a longer time to metastasis. In this case, E2 may trigger this effect independent of its receptor [30] or through other receptors where E2 is the ligand such as G-protein-coupled transmembrane receptor 30 (GPR30) [33] or truncated-ER [34], hypothesis that must be tested. 

In addition to prognostic value, ERα expression would allow to predict a response to anti-estrogenic hormone therapy. Thereby, patients with ERα-positive tumors may benefit from estrogen ablation (surgical sterilization) or ERα pharmacological blockage [35]. In women with breast cancer, the decision to implement hormone therapy anti-ERα depends on the expression of this receptor in the neoplastic tissue [29].

In relation to another ER (β isoform), there is a scarce evidence about its expression profile in CMTs. It has been described that approximately 30% express ERβ, which is higher in benign than in malignant masses. In malignant cases, its expression occurs predominantly in complex and mixed histologic subtypes [36]. Higher expression of ERβ has been also found in inflammatory mammary carcinomas compared to other mammary carcinomas [37]. Thereby, ERβ expression could have a dual role in mammary cancer, given its presence in benign tumors and high malignant neoplasms, nevertheless, these observations should be confirmed.

Due to its pleiotropic character, E2 can act as a mammary carcinogenic agent in humans and animals [38] by inducing, in association with its receptors, the transcription of several target genes [28]. Several clinical and experimental studies have confirmed that E2-activated ERα play a key role in mammary tumor promotion and progression [10,30,39], unlike the β-isoform, whose role in the pathogenesis of this disease should be elucidated [40].

Mechanisms of carcinogenic initiation associated with E2 include the generation of metabolites with genotoxic capacity derived from its oxidative metabolism [41]. E2 stimulates cell proliferation, increasing the possibility of the appearance of genetic errors and enhancing the proliferation of cell clones carrying potentially oncogenic mutations [42]. Thus, studies in mice have shown that the hydroxylated metabolites of the A ring of E2 (2 and 4 catechol-E2 and derivatives) can be carcinogenic in various tissues, including the mammary gland [41]. On the other hand, E2 3,4 quinone derivative can form unstable adducts that react with the bases adenine and guanine inducing depurinations and mutations. [41] To date, there are no studies in dogs that robustly demonstrate that E2-metabolites contribute to the development of mammary cancer. Nevertheless, as described in humans, there are evidence that support the idea that E2 would participate in tumor initiation. Firstly, this evidence is based on the high serum and mammary E2 concentrations in dogs with tumors relative to animals without mammary pathology. In this regard, it has been widely described significant high levels of 17β-E2 in serum of dogs with benign and malignant neoplasms in relation to normal mammary tissue [43,44,45,46]. The same does not happen with progesterone, whose serum levels do not change according to the pathological nature of the mammary tumors [43]. Secondly, there are some studies that have related the risk of developing the disease with the polymorphism of genes that code for enzymes involved in the metabolism of E2. This polymorphism is responsible for the inter-individual variability, modulating the exposure of mammary tissue to E2, influencing as a consequence, their susceptibility to mammary cancer [47]. In this context, the existence of SNPs of catechol-O-methyltransferase (COMT), an enzyme that participates in the inactivation of potentially genotoxic E2 metabolites, have been described in dogs, although this has not been shown to be a robust determining factor in canine mammary tumorigenesis [6]. Canada et al. [6] have recently defined associations between some COMT SNPs and pathological variables such as histological grade and vascular invasion. For this, 3 genotypes were established according to the presence of certain SNPs (Genotype 1: rs853046495; Genotype 2: rs23350589, rs23322686, rs23336579, rs852564758); Genotype 3: rs851328636 and rs853133060). Genotypes 1 and 3 were associated with low grade carcinomas, while Genotype 2 was related to intermediate/high grade carcinomas [6]. These results suggest that some COMT polymorphisms could be involved in neoplastic induction and progression, however, it is necessary to carry out novel studies in order to elucidate the underlying mechanisms.

Frequently, mammary tumors retain activities present in the previously normal mammary tissue; in this respect and as already described, it becomes particularly important the regulation of cell proliferation by E2 [39]. In estrogen-responsive mammary cancer cells, a variety of processes are regulated after the binding to of E2 to cognate intracellular receptors, which are localized in several target tissues and in different subcellular locations (cytoplasmatic, cell membrane and nucleus) [29,34]. These receptors have similar affinities for the ligand and are encoded by different genes [48]. ERα and ERβ belong to the superfamily of steroidal receptors, formed by a large group of transcriptional regulators, including ERs, progesterone receptors (PR), androgen receptors (AR), glucocorticoids receptors (GR), among others [34]. 

ERα protein structure comprises 5 domains: N-terminal domain (NTD or A/B), DNA-binding domain (DBD or C), Hinge region (D), ligand-binding domain (LBD or E), and C-terminal domain (F), whose molecular weight is 66 kDa [48]. The identity percentage between human and dog ERα is 94.6% with a length of 595 and 596 amino acids respectively [48], which allows us to extrapolate that most of the cellular and molecular processes associated to ERα activation described in humans, could also occur in dogs. Moreover, truncated-ERα have been described in breast cancer cell lines [34], which could partly mediate the biological effects of E2, especially in ER-negative tumor tissues. 46-kDa and 36-KDa ERα are frequently expressed at the extra-nuclear level, being codified by alternative splicing of the *ESR1* gene [34]. To date, their potential pathological role has not been clarified yet and neither in the context of CMTs.

Other ligands to ERα have been defined, called selective estradiol receptor modulators (SERM), which can act as agonists or antagonists of ER. This interaction can stimulate or block receptor function depending on the gene, cellular and tissue context. In human breast cancer, some SERMs such as tamoxifen or raloxifene are used as a therapeutic strategy given their antagonistic effect on the ERα [29]. However, in dogs, similar results have not been observed, probably due to a lower affinity for the receptor [48] and the induction of a powerful agonist effect in the endometrium [49].

### 2.1. Genomic Effects Associated with Estradiol

**Figure 1:**
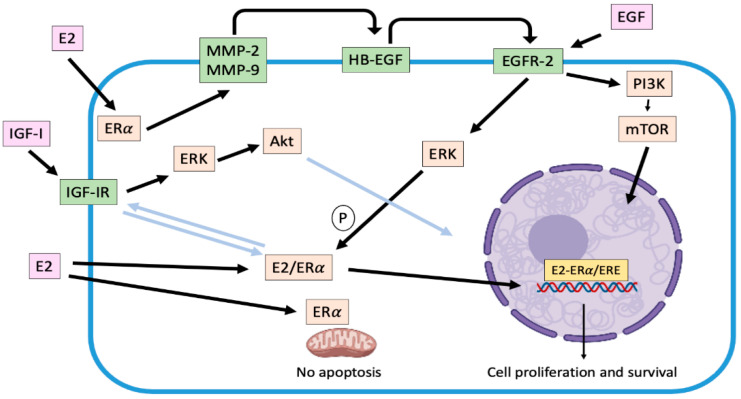
Representation of genomic and main non-genomic mechanisms associated with ERα activation in canine mammary carcinoma cells. Once activated by estradiol, ERα translocates from cytoplasm to nucleus, where it stimulates the transcription of several target genes (genomic effect). On the other hand, cross-talk between ERα and some growth factor receptors as EGFR-2 and IGF-IR are activated (non-genomic effects), which enhances the carcinogenic effects of the hormone. Light blue arrows indicate unconfirmed effects in canine mammary carcinoma cells. E2: estradiol; ERα: estradiol receptor α; IGF-I: insulin growth factor I; IGF-IR: insulin growth factor I receptor; MMP-2/9: matrix metalloproteinases 2/9; EGF: epidermal growth factor; HB-EGF: heparin binding epidermal growth factor; EGFR-2: epidermal growth factor receptor 2; PI3K: phosphatidylinositol 3-kinase; mTOR: mammalian target pf rapamycin; ERK: extracellular-responses kinases; Akt: protein kinase B; ERE: estrogen response elements; P: phosphorylation.

It has been widely described that the subcellular distribution of ERα is modified in response to E2 [34]. The hormone binding induces a receptor translocation from the cytoplasm to the nucleus. Cytoplasmic ERα is associated with cytoskeletal elements, through a region of 70 amino acids located at the carboxylic end of domain E and part of domain F [29], which would facilitate its translocation. When ER are not bound by E2, their transcriptional activity is off due to their association with heat shock proteins. When binding to ligand, ER will dissociate from the heat shock proteins and undergoes a conformational change, phosphorylation, and nuclear translocation [50]. Thus, association of E2 with nuclear ERα modulates gene transcription (Figure 1). This regulation can be the product of the direct binding of dimerized E2-ER complexes to estrogen response elements (ERE) at the promoter sites of target genes [34]. These ERE are a palindromic consensus sequence of AGGTCA motifs separated by a 3-base spacer. E2-ER complex is associate with transcriptional coactivators and, subsequently, recruit RNA polymerase and general transcription factors [50]. The coactivator molecules consist of a protein complex with chromatin remodeling activity that promotes the recruitment of transcription factors. By contrast, when some SERM with antagonist activity binds to the ER, corepressors are recruited and form a complex with histone deacetylases maintaining the structure of chromatin stiff [50]. Furthermore, nuclear ERs can be indirectly associated with DNA, through protein-protein interactions with other transcription factors such as AP-1 and Sp-1 [29,50]. As a result of these interactions, coactivator recruitment (and co-repressor displacement) is induced at DNA binding sites, thus modulating gene expression and consequent protein expression [51]. Many of these genes directly promote cell proliferation, survival, differentiation and tumor progression [28]. Stand out among others, the insulin growth factor receptor (IGFR), the cell cycle regulator Cyclin D1, the anti-apoptotic factor Bcl-2 and the vascular endothelial growth factor (VEGF) [50,51].

### 2.2. Non-Genomic Effects Associated with Estradiol

Several non-genomic effects are also regulated by the association of E2 or SERMs to estrogen receptors in target cells. This mechanism is also known as "membrane-initiated steroidal signaling (MISS)" [51]. These responses can occur in seconds (calcium flux, cyclic nucleotide induction) or minutes (kinases activation), triggering post-translational modification of several proteins, especially through phosphorylation processes, and do not result from immediate regulation of gene transcription [51]. In fact, this signaling is more related to the location in the cell membrane and cytoplasm of the ERα and not to its nuclear location. ERs are arranged in caveolar vesicles across the plasma membrane in a similar manner to receptors for various growth factors and are assembled as part of a large complex that includes tyrosine-kinases receptors (EGFR/HER-2, IGFR, among others), tyrosine-kinases molecules like Src, and some G protein-coupled receptors. In this regard, in domain E there is a sequence of residues that participates in binding of ER to the plasma membrane, allowing the ERα to cross-talk with EGFRs [52,53]. These non-genomic effects contemplate the modulation (activation or release) by the E2/ERα complex of various regulatory proteins located in the cytoplasm or associated with cell membrane such as mitogen-activated kinases (MAPK), phosphatidyl inositol 3 kinase (PI3K), protein kinase C (PKC), cyclic adenosine monophosphate (cAMP) and calcium, among others [53].

One of the main non-genomic mechanisms described by which E2 promotes tumor progression in ER-positive human [28,53] and canine [39] mammary tumor cells, is the activation of matrix metalloproteinases MMP-2 and 9 in response to 2 nM of the hormone. Their activation, mediated by α subunits of G-protein-coupled receptors and Src, cleave and release HB-EGF, a factor capable of binding and activating EGFR-2 resident in the plasma membrane. Consequently, a time-dependent increase in extracellular-response kinases (ERK) phosphorylation is induced, triggering a greater proliferative activity [28,39,53] (Figure 1). Moreover, phosphorylation of ERK 1/2 (p44MAPK and p42MAPK respectively) can trigger phosphorylation in serine 118 of the domain A/B of the ERα enhancing its genomic activity [52]. In this context, it is possible to describe that both genomic and non-genomic mechanisms are not exclusive [51]. Once EGFR is activated, it dimerizes [28,39] and translocates to the nucleus, upregulating the expression of genes such as Cyclin D1 [54]. On the other hand, EGFR can upregulate nuclear-ERα, independent of E2, inducing its phosphorylation and activation, mediated by ERK activation [53]. Thus, this bidirectional relation or cross-talk between ERα and EGFR is configured, enhancing tumor progression. In addition, activation of EGFRs upregulates the synthesis and secretion of different pro-angiogenic growth factors such as VEGF, interleukin 8 (IL-8) and basic fibroblast growth factor (bFGF) [54]. These data suggest that the EGFRs system (that include its relation to ERα-activation) is an important mediator of autocrine and paracrine circuits that result in angiogenesis and enhanced tumor growth [29].

Other non-genomic mechanism stablished is the interaction between ERα and IGFR, which has been described in ERα-positive human mammary tumor cells, where both E2 and IGF-1 induced the interaction ER-IGF-1R enhancing cell proliferation. Moreover, activated ERα trigger IGF-1 signaling pathway mediated by activation of ERK 1/2 and Akt [55]. In the CMT context, Queiroga et al. [56] have described that E2 could upregulate the IGF-1 mammary production. This situation would occur in malignant tumors, where there is a low ERα expression and a high expression of IGF-1R [57].

The genomic and non-genomic pathways associated with E2 interact at different levels with kinase networks (dependent or not on growth factors). These interactions exacerbate different bidirectional signaling pathways, important in the pathogenesis of this disease, playing a fundamental role in the promotion and progression of mammary tumors, as well as in the potential development of resistance to endocrine therapy.

Moreover, the presence of ERα at the mitochondrial level has been described in mammary tumor and in endothelial cells, where its activation inhibits both the release of cytochrome C induced by UV radiation and the mitochondrial membrane potential, events that are directly related to the intrinsic pathway of apoptosis [58]. Furthermore, E2 inhibits the formation of reactive oxygen species (ROS) in the mitochondria through rapid activation of the superoxide dismutase (SOD). In this way, the steroid favors the survival of tumor cells [34,58].

A distinct receptor than canonical ERs has been described, which can bind E2. This protein is GPR30, also known as GPER-1, that locate on the plasma membrane and endoplasmic reticulum membrane. Once activated, different non-genomic effects are triggered such as the mobilization of intracellular calcium and the activation of ERK 1/2, contributing to the pathophysiological mechanisms induced by the hormone. One of these mechanisms also involves the activation of MMP-2 and 9 with the subsequent secretion of HB-EGF from the plasma membrane, activating EGFR. This mechanism could provide a possible explanation for the estrogen-induced mitogenic effect in human breast cancer cells in which ER is not detectable [33,59]. GPR30 expression has not been studied in CMT. It is therefore relevant to define what would be the function of this receptor in this pathology and if it participates in the activity of estradiol in tumors that lack the canonical ER expression. Some studies have been done on the expression of GPR30 in human mammary neoplasms. In this regard, Filardo et al. [60] studied REα and GPR30 expression in breast cancer through immunohistochemistry, observing that of a total of 321 tumor tissues analyzed, approximately 60% expressed only GPR30 and 40% both receptors. This co-expression could indicate an association between both receptors, which is not yet well known [60]. In this regard, GPR30 activation could imply an alternative and independent pathway to canonical ERs by contributing to the biological effects of E2 and EGFR on tumor progression. This would explain, at least in part, why ER-negative tumors exhibit a longer disease-free time in presence of high serum concentrations of the hormone [30]. Moreover, it has also been defined that in ER-negative tumors, EGF expression would upregulate the expression of GPR30, stimulating tumor cells growth [61]. GPR30 is positively associated with tumor size, presence of metastasis, high HER-2 expression [59], and drug resistance [62], whereby, its expression could be considered as a predictor of poor prognosis. These antecedents reinforce the concept that both receptors, REα and GPR30, respond to different biological influences on the growth and progression of mammary tumors, which must be analyzed in detail in CMTs.

### 2.3. Intracrinology of Estradiol in Canine Mammary Tumors

**Figure 2:**
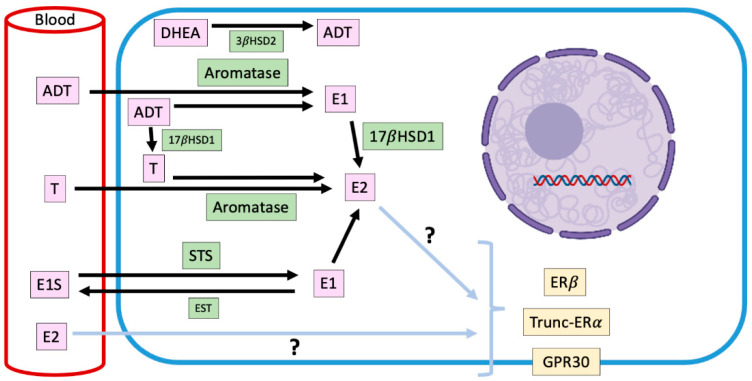
Representation of intracrine estradiol synthesis and its main precursors in ERα-negative canine mammary tumors. Mammary estradiol production in ERα-negative canine mammary tumors, where the responsible steroidogenic enzymes, their substrates and products, and the receptors that could mediate its pro-carcinogenic effects are shown. E2: estradiol; T: testosterone; E1: estrone; E1S: estrone sulfate; DHEA: dehydroepiandrosterone; ADT: androstenedione; STS: steroid sulfatase; EST: estrogen sulfotransferase; 17βHSD1: 17β-hydroxysteroid dehydrogenase type 1; 3βHSD2: 3β-hydroxysteroid dehydrogenase type 2; ERα: estradiol receptor α; ERβ: estradiol receptor β; GPR30: G-protein-coupled transmembrane receptor 30.

Endocrine signaling is made up of glandular tissue that synthesizes hormones that are transported through the bloodstream to trigger their biological effect on a specific target organ. A variant of this is paracrine and autocrine signaling [63]. There is also a mechanism that has been called intracrine, in which the hormone also exerts its effect in the place where it was synthesized, but inside the cell [63]. In mammalian females, the main source of estrogens is the ovary, nevertheless, it has described a peripherical synthesis of estrogens in normal and tumor mammary tissues [43]. There is evidence to suggest that local production of steroids has a more relevant influence on the development of mammary cancer than circulating estrogens, which would contribute to the maintenance of high levels of these hormones in mammary tissue [43]. It has been established that in dog females with mammary tumors, high concentrations of steroidal hormones are locally produced unlike what happens in animals without mammary pathology [43,44,45,64], being in some cases, higher concentrations in neoplastic tissue than in serum [46] (Figure 2). These local steroids production is positively correlated with EGF secretion [64], which suggests that E2 and the growth factor are interacting within mammary microenvironment. The highest production of steroids occurs in malignant tumors, including inflammatory mammary carcinomas, where this synthesis tends to be even higher [46]. These hormones include estrogens as 17β-E2 and estrone sulfate, and androgens as androstenedione, dehydroepiandrosterone, and testosterone and their local production (Figure 2).

In the mammary gland, the local synthesis of estrogens is carried out by steroidogenic enzymes such as CYP19-Aromatase, 17-β-hydroxysteroid dehydrogenase type I (17βHSD1) and steroid sulfatase (STS), whose expression have been documented in CMTs, although, without prognostic significance [43].

Aromatase is an enzyme that is located in the endoplasmic reticulum of adipocytes, tumor-associated fibroblasts and mammary cancer cells and catalyzes the aromatization of androstenedione to estrone, and testosterone to E2 [65]. The expression and activity of this enzyme are increased in mammary malignant tumors; therefore, its presence could be involved in the progression of the disease [43]. Lim et al. [66] have informed that mammary aromatase expression is significantly higher in obese and overweight dogs, condition that could trigger the higher intratumor production of E2 already described, and explain, in part, the risk that its presence implies in the development of mammary tumors. 

Estrone-sulfate is a biologically inactive form of estrogen, which makes up the largest amount of circulating estrogen in plasma [67]. STS is the enzyme responsible for hydrolyzing estrone sulfate to estrone. In humans, STS is expressed mostly in tumor than normal tissues [68], although its impact on clinicopathological variables with prognostic value is not clear [68,69]. In dogs, there is only one report regarding the expression of STS in mammary tumors, which diminishes in malignant tumors in relation to normal mammary tissue [67], which suggests that the mammary levels of estrone would be explained fundamentally by the enzymatic activity of aromatase on androstenedione.

On the other hand, 17βHSD1 catalyze the reduction of estrone to E2. In humans, this enzyme would be important in the pathogenesis and progression of estrogen-dependent mammary cancer [70], however, in dogs, its mammary expression has not been described yet. 

Considering the above, it is necessary to stablish the exact role of these steroidogenic enzymes on tumor progression and prognosis.

## 3. Future Perspectives

Uptake from circulation and/or a local steroid production provide target cells with endogenous estrogen for the activation of their ER. In this context, further knowledge is required on the activation of ERα signaling pathways by several compounds in mammary cancer cells. On the other hand, it is relevant to define how E2 could induce its pro-carcinogenic role within mammary tumor niche, considering that the highest production of the steroid occurs in malignant tumors, where a high proportion of them do not express ERα. Perhaps, GPR30, truncated ERα, ERβ in inflammatory mammary carcinomas, or ER-independent outcomes could mediate these effects, which are hypotheses that must be analyzed in future studies. Moreover, certain evidence indicates that the study of the tumor microenvironment should be a target due to the importance of intracrine estrogens production and the scarce information about it in canine mammary tumors.

## 4. Conclusions

Most of the studies on estradiol in canine mammary cancer have focused on the tissue expression of ERα and its potential prognostic value, scarcely exploring the mechanisms underlying the effect of the steroid on tumor cells. This review discussed some of the molecular mechanisms involved in the action of this hormone that promote carcinogenic initiation, promotion, and progression, however, many of these mechanisms remain incompletely elucidated. Within of these mechanisms, emphasis was placed on the genomic and non-genomic mechanisms linked to the activation of ERα by estradiol, in order to understand the way in which the hormone exerts its pleiotropic action and to infer potential mechanisms of resistance to blocking ERα activation.

## Data Availability

No new data were created in this study. Data sharing is not applicable to this manuscript.
